# Effects of transcranial combined with peripheral repetitive magnetic stimulation on limb spasticity and resting-state brain activity in stroke patients

**DOI:** 10.3389/fnhum.2023.992424

**Published:** 2023-04-04

**Authors:** Yin Qin, Xiaoying Liu, Yinxin Zhang, Jiwei Wu, Xiaoyang Wang

**Affiliations:** ^1^Department of Rehabilitation Medicine, The 900th Hospital of People’s Liberation Army (Fuzhou General Hospital of Nanjing Military Region), Fuzhou, Fujian, China; ^2^Department of Radiology, The 900th Hospital of People’s Liberation Army (Fuzhou General Hospital of Nanjing Military Region), Fuzhou, Fujian, China

**Keywords:** stroke, upper limb spasticity, repetitive transcranial magnetic stimulation (LF-rTMS), peripheral repetitive magnetic stimulation, amplitude of low-frequency fluctuation (ALFF)

## Abstract

**Background and objective:**

Transcranial magnetic stimulation and peripheral repetitive magnetic stimulation (rPMS), as non-invasive neuromodulation techniques, can promote functional recovery in patients with post-stroke spasticity (PSS), but the effects of transcranial magnetic stimulation combined with peripheral magnetic stimulation on PSS remain largely unknown. Therefore, we examined the effects of low-frequency repetitive transcranial magnetic stimulation (LF-rTMS) combined with rPMS on PSS patients and its potential neural correlates to behavioral improvements.

**Methods:**

Forty-nine PSS patients were divided randomly into three groups: a combined group (*n* = 20), a LF-rTMS group (*n* = 15), and a control group (*n* = 14). The combined group received LF-rTMS and rPMS treatment, the rTMS group received LF-rTMS treatment, and the control group received only routine rehabilitation. All patients underwent Ashworth Spasm Scale (MAS), upper extremity Fugl-Meyer (FMA-UE), and modified Barthel Index (MBI) assessments before and after intervention. In addition, resting-state functional magnetic resonance imaging data were collected pre- and post-treatment to observe changes in the amplitude of low-frequency fluctuation (ALFF).

**Results:**

The MAS score was decreased, FMA-UE score and MBI scores were increased in the three groups after therapy than before therapy (all *P* < 0.05). In particular, the combined group showed significant effect on improved motor function and relieved spasticity in PSS (*P* < 0.01). Moreover, the combined treatment increased ALFF values mainly in the right supplementary motor area, right middle frontal gyrus, and right cerebellum, while reduced ALFF values mainly in the right post-central gyrus compared with pre-treatment. Compared with the LF-rTMS and control groups, the combined treatment increased ALFF values in the right cerebellum and reduced ALFF values mainly in the frontoparietal cortex. Improvements in the MAS score were positively correlated with the change in ALFF values in the right cerebellum (*r* = 0.698, *P* = 0.001) and the right supplementary motor area (*r* = 0.700, *P* = 0.001) after combined treatment.

**Conclusion:**

Transcranial combined with peripheral repetitive magnetic stimulation could improve spastic state and motor function in PSS patients, and this effect may be associated with altered cerebellar and frontoparietal cortical activity.

**Clinical trial registration:**

http://www.chictr.org.cn/index.aspx, identifier ChiCTR1800019452.

## Introduction

Stroke is characterized by high morbidity, disability and recurrence rates (Wu et al., 2019). About 20–40% of stroke survivors develop muscle spasticity problems, which leads to disability in 70–80% of them, resulting in loss of ability to work and daily living (Thibaut et al., 2013; Hsu et al., 2022). In particular, patients with severe spasticity also develop deforming changes in muscles, bones, and joints, making it necessary for the patient’s family and society to invest a lot of energy and financial resources in the care and treatment of the patient (Cabanas-Valdés et al., 2020; Lackritz et al., 2021). For a long time, upper limb spasticity has been the difficulty in rehabilitation treatment for the dysfunction of patients with subacute and chronic stroke (Gittler and Davis, 2018). The common methods for reducing patients’ muscle spasticity include rehabilitation therapy, electrical stimulation, drug therapy, botulinum toxin A, and surgery (Hu et al., 2021; Xu D. et al., 2021; Hsu et al., 2022). However, these therapies have limitations to different degrees, and a single treatment regimen does not achieve satisfactory effects. As a result, there is an urgent need to develop new interventions.

Low frequency repetitive transcranial magnetic stimulation (LF-rTMS), as a non-invasive neuromodulation technique, is an important method for inducing plasticity in the central nervous system (Li Y. et al., 2020). It uses electromagnetic induction to induce physiological changes that can regulate brain activity and promote cortical functional remodeling to improve motor function and spasticity after stroke (Luk et al., 2022). For example, studies have shown that LF-rTMS can reduce muscle spasticity by activating the central nervous network and reconstructing central control (Volz et al., 2016; Gottlieb et al., 2021). In addition, rPMS is a recently discovered new treatment modality that excites nerves or muscles by giving repetitive, high-frequency, high-intensity magnetic fields to tissues outside the brain. RPMS can stimulate peripheral proprioceptive afferent nerves, increase proprioceptive and somatosensory input, improve the plasticity of the damaged nervous system, and rebuild normal reflexes and control (El Nahas et al., 2022). RPMS has been shown to improve motor control in stroke patients (Chen et al., 2020). Recently, some scholars have proposed that transcranial combined with peripheral repetitive magnetic stimulation may produce better therapeutic effects than single object stimulation (Yang et al., 2022). However, to our knowledge, whether the combination of LF-rTMS and rPMS improves the therapeutic effectiveness in PSS patients remains uncertain, and its potential neurological relevance has not been reported.

Resting-state functional magnetic resonance imaging (rs-fMRI) is a strong non-invasive tool for evaluating brain function by discovering proportional changes in the local deoxyhemoglobin content when assessing neuronal activity (van Hees et al., 2014). Analyses of the amplitude of low-frequency fluctuations (ALFF) is a crucial method employed in rs-fMRI studies to detect the intensity of spontaneous neural activities in BOLD signals (Li G. et al., 2020; Chen et al., 2021). ALFF is currently widely used to investigate the neurobiological mechanisms of depression, stroke, Parkinson’s disease, and other diseases (van Hees et al., 2014; Teng et al., 2018; Yadav et al., 2018), but it has not been employed in the treatment and evaluation of PSS. Therefore, using this indicator can help us to determine the pathophysiological features of PSS and understand the brain effect mechanism after rTMS combined with rPMS intervention.

Therefore, we used the modified Ashworth Spasm Scale (MAS), upper extremity Fugl-Meyer scale (FMA-UE), and modified Barthel Index (MBI) to investigate the clinical efficacy of LF-rTMS combined with rPMS treatment. In addition, ALFF analyses were performed to explore the potential neural correlation with behavioral improvements following LF-rTMS combined with rPMS treatment in PSS patients. We hypothesized that LF-rTMS combined with rPMS could improve the spastic state and motor function of PSS patients, which may be related to the altered intensity of spontaneous neural activity in some brain regions.

## Materials and methods

### Trial design and subjects

**FIGURE 1:**
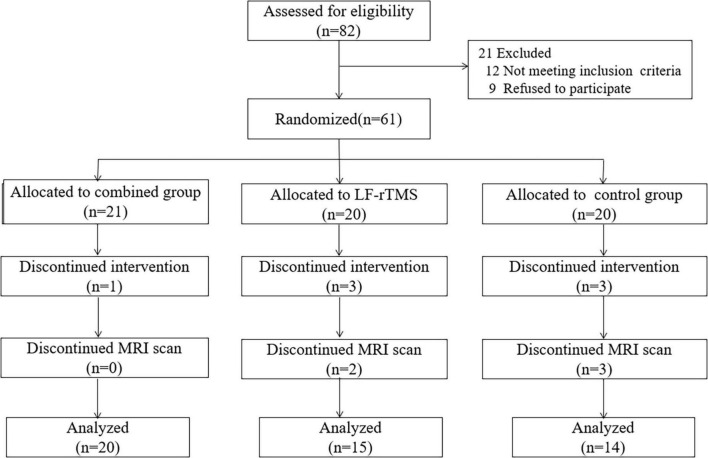
The flow charts of the research design.

This single-center randomized clinical study evaluated LF-rTMS combined with rPMS effects on ischemic stroke patients with upper limb muscle spasticity (Figure 1). The present study trial was approved by the ethics committee of the 900th Hospital of People’s Liberation Army (No. 2015011), and written informed consent was provided by each study participant. Inclusion criteria for this study were: (a) first-ever ischemic stroke, (b) between 35 and 75 years of age, (c) right-handedness before stroke, (d) disease course between 1 and 6 months, (e) lesion in the middle cerebral artery territory, (f) upper limb Brunnstrom recovery period of 3–5, (g) upper spasticity MAS score of 1–3. The exclusion criteria for this study were: (a) a history of recurrent stroke, (b) other abnormal brain findings on CT or MRI images, (c) other psychiatric or neurological conditions, (d) white matter hyperintensities greater than 1 (Fazekas et al., 1987), (e) injections of botulinum toxin A in the affected upper extremity within the last 6 months, and (f) taking anti-spasmodic medications within the last month. Sixty-one stroke patients with upper limb spasticity agreeing to take part in the study were assigned to three groups randomly: the combined group (*n* = 21), the single LF-rTMS group (*n* = 20), and the control group (*n* = 20). As a result of microparticle shedding during treatment or the absence of MRI scans, eighteen patients were excluded from the study. Finally, forty-nine stroke patients were enrolled, including 20 patients (mean age: 60.05 ± 9.97 years) who underwent LF-rTMS combined with rPMS treatment, 15 patients (mean age: 55.87 ± 10.50 years) who underwent single LF-rTMS treatment, and 14 patients (mean age: 59.43 ± 9.12 years) who underwent conventional rehabilitation.

### Rehabilitation intervention

All patients were given routine rehabilitation training. Routine rehabilitation training included good body posture, passive joint motion, muscle stretching exercises, and neurodevelopmental therapy; each training period lasted 40 min.

For patients in the combined group and LF-rTMS group, we measured the resting motor threshold (RMT) before stimulation, which corresponded to the lowest rTMS intensity capable of eliciting a motor-evoked potential >50 μV in at least five of ten consecutive stimulations over the primary motor area (M1) (Bolton et al., 2012; Guo et al., 2021).

The combined group was given LF-rTMS and rPMS treatment. ➀ LF-rTMS treatment: LF-rTMS was performed using a transcranial magnetic stimulator (MAGSTIM, UK) equipped with an air-cooled figure-eight focal coil. Prior to treatment, the optimal target of stimulation in the M1 region of the unaffected hemisphere should be located. The patient lies flat on the treatment bed in a supine position with the whole body relaxed and the head immobilized. The surface electrode is placed on the abdominal muscle of the abductor pollicis brevis in the unaffected side hand of the patient. A transcranial magnetic cap was used to locate representative areas of the upper motor cortex. The rTMS stimulation frequency was set at 1 Hz, and an intensity level of 90% RMT was used. Ten pulses were delivered in each sequence with an interval of 2 s, and a total of 1,200 magnetic pulses were delivered. ➁ RPMS treatment: Peripheral magnetic stimulation was performed immediately after the completion of LF-rTMS. RPMS also adopted a magnetic stimulator with an “8” coil and the stimulation site was Erb’s point of the upper limb on the affected side. The motion threshold of the peripheral magnetic stimulation was defined as the minimum intensity of stimulation that can cause subtle visible contractions in the bunion and muscle eyes of the affected side. The intensity of stimulation was appropriate for the patient’s muscle contraction. In total, 10 Hz stimulations were delivered in 3 s sessions with an 8 s interval between sessions for a total of 1,200 pulses per treatment.

In the LF-rTMS group, only LF-rTMS stimulation was given, and the stimulation target and stimulation protocol were the same as those in the combined group. The control group was given conventional treatment only, and did not receive magnetic stimulation treatment.

All stroke patients were treated one a day, 5 days a week, for 8 weeks.

### Neuroimaging data acquisition

MRI data of all subjects were acquired before and after treatment employing a 3-Tesla Siemens scanner with a 12-channel head coil. Six-minute resting-state MRI scans were performed, during which participants were asked to lie quietly, stay awake, and think about nothing. The functional images of subject were acquired using the gradient echo planar imaging (EPI) sequences: repetition time = 2000 ms, echo time = 21 ms, acquisition matrix = 64 × 64, field of view = 240 × 240 mm^2^, slice number = 33, slice thickness = 4 mm, and slice gap = 0.8 mm. The following sequences were used to acquire high-resolution 3D-T1 images: repetition time = 1900 ms, echo time = 2.52 ms, acquisition matrix = 256 × 256, field of view = 240 × 240 mm^2^, slice thickness = 1.0 mm, and no gap. In addition, T2FLAIR and DWI were routinely applied to exclude other brain diseases.

### Behavioral assessments

Behavioral evaluations of PSS patients were performed by the same senior rehabilitation therapist blinded before and after the interventions. The MAS score was used to assess muscle tension (Bohannon and Smith, 1987). For convenience, grades 0, I, I+, II, III, and IV are converted into 0, 1, 1.5, 2, 3, and 4 for statistical analysis. Higher MAS scores mean more severe spasticity. The Meyer upper extremity assessment scale (FMA-UE) was employed to assess upper limb motor function with an overall score of 66 points (Mooney et al., 2019). The higher FMA-UE score, the better motor function of the limbs. The modified Barthel Index (MBI) with a full score of 100 points was applied to assess PSS patients’ self-care ability in their daily life (Ohura et al., 2017). The higher MBI scores, the better ability of daily living activities.

### Data preprocessing

The preprocessing of rs-fMRI data and statistical analyses were performed using RESTplus^1^ and SPM12.^2^ Prior to data preprocessing, we flipped the image data of the left hemispheric lesion from left to right to ensure that the left hemisphere was the lesion side. The preprocessing steps included discard five initial time points of each participant, slice timing, and head motion correction. Patients were excluded when head movement was greater than 2 mm or 2°. However, the data were not excluded from this study. Subsequently, the deformation fields produced from tissue segmentation of structural images were then used to normalize functional images into the Montreal Neurological Institute (MNI) space and resample to 3 × 3 × 3 mm^3^ voxels. Next, the images were smoothed with a full-width of 6 mm under half-maximum Gaussian kernel. Finally, physiological high-frequency noise and low-frequency drift were removed by detrending and bandpass filtering (0.01−0.08 Hz).

### ALFF analysis

RESTplus software was used for ALFF. The ALFF calculation method has been introduced in previous related studies (Su et al., 2019; Machner et al., 2020). A bandpass filter of 0.01∼0.08 Hz was adopted to decrease the influence of high-frequency noise and low-frequency drift. The filtered time series was converted into the frequency domain using the quick Fourier transform. The average square root of the power spectrum, which was taken as the ALFF value, was determined after acquiring the power spectrum. After subtracting the average value and dividing by the whole brain voxel deviation, the ALFF value was converted to z-distribution to achieve standardization. Each participant’s Z-ALFF atlas was used for further intergroup statistical analysis.

### Statistical analysis

Statistical analyses used SPSS 23.0 software (IBM, Corp, Armonk, NY, USA). The measurement data were reported as mean ± standard deviation when data follow a normal distribution. Demographic information and clinical characteristics were compared among the three groups using one-way analysis of variance followed by Bonferroni’s *post-hoc* test. The chi-square test was employed for sex comparisons, and the paired *T*-test was employed for intragroup comparisons before and after treatment.

RESTplus software was used for one-way ANCOVA to compare ALFF image differences among the combined treatment group, single LF-RTMS group and control group. Subsequently, *post-hoc* analysis was used to compare the differences between each pair of groups. Paired-sample *t*-tests were used to examine differences in ALFF before and after treatment. Gender, age, and years of education were used as covariates to decrease their possible influence. All statistical maps were rectified for multiple comparisons using AlphaSim rectification with a *P*-value < 0.001.

In addition, Pearson correlations were calculated to analyze the association between MAS score improvement and changes in ALFF values. A value of *P* < 0.05 was considered to demonstrate statistically significant differences.

## Results

### Baseline characteristics

In total, 20 PSS patients were treated with combined treatment (11 males; mean age, 60.05 ± 9.97 years), 15 PSS patients were treated with single LF-rTMS (9 males; mean age, 55.87 ± 10.50 years), and 14 PSS patients were treated with routine rehabilitation training (11 males; mean age, 59.43 ± 9.12 years). No significant differences in sex, age, education level, or disease period were noted among the three groups. Regarding clinical data (all *P* > 0.05), no significant difference in FMA-UE score, MAS score, or MBI score were noted among the single LF-rTMS group, combined treatment group, and control group before treatment (all *P* > 0.05) (Table 1).

**TABLE 1:** Baseline characteristics of stroke patients.

Variables	Combined group (*n* = 20)	LF-rTMS (*n* = 15)	Control group (*n* = 14)	F/χ^2^	*P*-value
Age, year	60.05 ± 9.97 (37−74)	55.87 ± 10.50 (39−73)	59.43 ± 9.12 (42−72)	0.857	0.431
Men, %	11 (55%)	9 (60%)	11(79%)	2.068	0.356
Education, year	9.8 ± 2.91	9.06 ± 3.78	9.28 ± 2.97	0.359	0.700
Disease duration (month)	3.45 ± 1.76	3.20 ± 1.93	2.85 ± 1.74	0.441	0.646
Lesion location				2.090	0.352
Left hemisphere	8(40%)	3(20%)	6(42%)		
Right hemisphere	12(60%)	12(80%)	8(58%)		
MAS	1.70 ± 0.44	1.76 ± 0.45	1.78 ± 0.51	0.163	0.850
FMA-UE	26.65 ± 8.89	25.33 ± 9.91	23.64 ± 7.61	0.774	0.467
MBI	40.35 ± 16.45	35.13 ± 16.25	32.14 ± 13.41	1.214	0.306

Data are presented as the means ± SDs (range) for continuous data and *n* (%) for categorical data. MAS, Modified Ashworth Scale; FMA-UE, upper extremity Fugl-Meyer Assessment; MBI, modified Barthel Index test.

### Analysis of clinical characteristics

**FIGURE 2:**
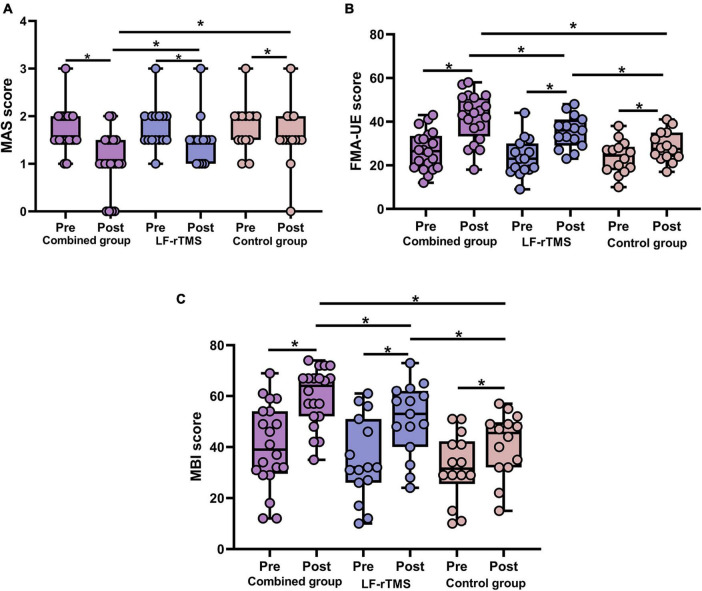
Behavioral changes in the combined group, LF-rTMS group and control group. **(A)** The MAS score was decreased post-treatment compared with pre-treatment in the three groups. After treatment, the combined group had a lower MAS score than the other two groups. **(B)** The FMA-UE score was higher post-treatment than pre-treatment in the three groups. The combined group had a higher FMA-UE score than the other two groups after treatment. **(C)** After treatment, the three groups showed improvements in the MBI. The MBI score was higher in the combined group compared with the control group and LF-rTMS after treatment. *P < 0.05. MAS, Modified Ashworth Scale; FMA-UE, upper extremity Fugl-Meyer Assessment; MBI, modified Barthel index; Pre, pretreatment; Post, post-treatment.

Muscle tone was evaluated using the MAS score (Table 2). Three groups showed significant improvements in the MAS score after treatment (all *P* < 0.05). In addition, MAS score among groups after treatment showed statistically significant differences (*F* = 4.766, *P* = 0.013). In particular, the MAS score of the combined treatment was markedly decrease than that of the LF-rTMS treatment (*P* = 0.045) and control group (*P* = 0.032). However, we found no significant difference in MAS score between the control group and the LF-rTMS treatment (*P* > 0.05) (Figure 2A).

**TABLE 2:** Comparison of clinical characteristics among the combined group, LF-rTMS, and control group.

	Combined group	LF-rTMS	Control group	*P*-value	*P*_combinedvs.LF–rTMS_	*P*_combinedvs.control_	*P*_LF–rTMSvs.control_
**MAS**							
Pre-treatment	1.70 ± 0.44	1.76 ± 0.45	1.78 ± 0.51	0.850	0.677	0.600	0.913
Post-treatment	1.03 ± 0.61*	1.53 ± 0.48*	1.57 ± 0.64*	0.013	0.045	0.032	0.862
**FMA-UE**							
Pre-treatment	26.65 ± 8.89	25.33 ± 9.91	23.64 ± 7.61	0.467	0.287	0.314	0.972
Post-treatment	41.90 ± 10.86*	35.26 ± 7.43*	28.57 ± 7.00*	0.0004	0.034	0.00009	0.049
**MBI**							
Pre-treatment	40.35 ± 16.45	35.13 ± 16.25	32.14 ± 13.41	0.306	0.332	0.138	0.608
Post-treatment	59.75 ± 11.43*	50.80 ± 14.27*	41.21 ± 12.49*	0.01	0.044	0.00012	0.047

Continuous data were expressed as means ± SDs. MAS, Modified Ashworth Scale; FMA-UE, upper extremity Fugl-Meyer Assessment; MBI, modified Barthel Index test. *Represents the difference before and after intervention.

The FMA-UE score was employed to assess upper limb motor function (Table 2). Compared with before treatment, the FMA-UE score in all three groups obviously improved after treatment (all *P* < 0.05). In addition, FMA-UE scores among groups after treatment showed statistically significant differences (*F* = 9.294, *P* = 0.0004), and the FMA-UE score of the combined treatment was markedly improved compared with the single LF-rTMS (*P* = 0.034) or control group (*P* = 0.00009) (Figure 2B).

The MBI score was used to assess the activities of daily living (Table 2). After treatment, MBI score of patients in three groups were improved compared with those before treatment (all *P* < 0.05). Moreover, there was a statistical difference in MBI score among the three groups after treatment (*F* = 8.891, *P* = 0.001). The MBI score of the combined treatment was significantly increased than that of single LF-rTMS (*P* = 0.044) or control group (*P* = 0.00012) after treatment (Figure 2C).

### Changes in ALFF

**FIGURE 3:**
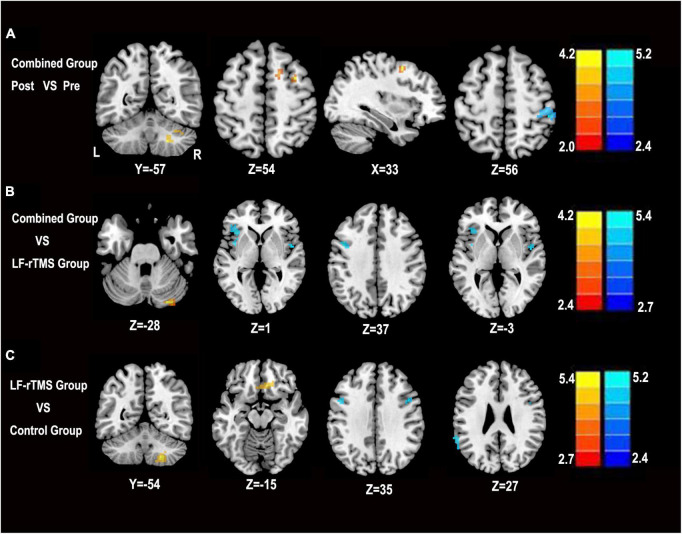
Statistical parametric map showing the significant differences in ALFF among the combined group, LF-rTMS and control group. **(A)** Change in ALFF in the combined pre-treatment group compared with the post-treatment group. **(B)** Differences in ALFF between the combined group and the LF-rTMS group. **(C)** Differences in ALFF between the combined group and the control group. The significance level was defined at *P* < 0.05 with AlphaSim correction. The color bar denotes the *t*-value. R, right; L, left, Pre, pre-treatment in the combined group; Post, post-treatment in the combined group.

We first performed comparisons in pre- and post-combined treatment to explore the effects of LF-rTMS combined with rPMS treatment in PSS. Compared with pre-treatment, the enhanced ALFF following combined treatment mainly included the right supplementary motor area (SMA), right cerebellar, and right middle frontal gyrus. In contrast, reduced ALFF value was found in the right postcentral gyrus (*P* < 0.05, AlphaSim corrected) (Figure 3A).

Then, we compared the combined group, LF-rTMS group, and control group to identify regions in the combined group that showed changes in ALFF values. Compared with the single LF-rTMS treatment, the combined treatment increased the ALFF values mainly in the right cerebellum and reduced the ALFF values mainly in the left precentral gyrus, left middle frontal gyrus, left inferior frontal gyrus, and right insula (*P* < 0.05, AlphaSim corrected) (Figure 3B). Compared with the control group, the combined treatment increased ALFF values mainly in the right cerebellum and right medial frontal gyrus. In contrast, a reduced ALFF value was found in the left precentral gyrus and left supramarginal gyrus (*P* < 0.05, AlphaSim corrected) (Figure 3C).

### Correlation analysis

**FIGURE 4:**
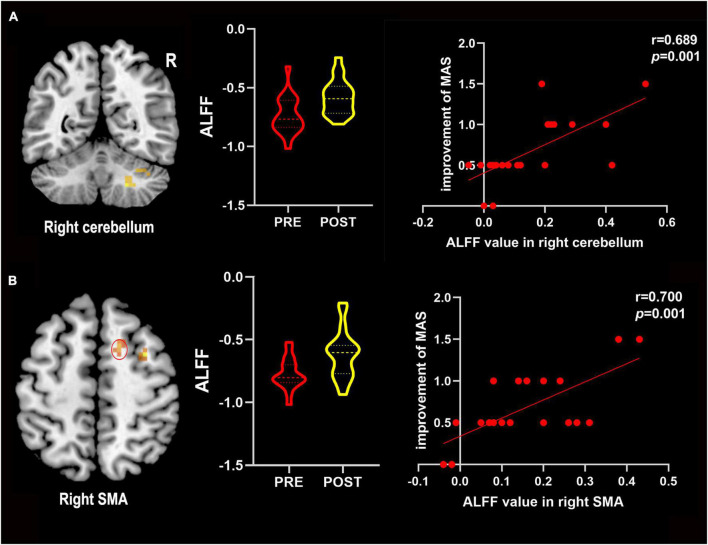
The change in ALFF values positively correlated with improved MAS scores. **(A)** Improvements in the MAS score positively correlated with the change in ALFF values in the right cerebellum. **(B)** Improvements in the MAS score positively correlated with the change in ALFF values in the right SMA. R, right; SMA, supplementary motor area; Pre, pre-treatment in the combined group; Post, post-treatment in the combined group.

Correlation analyses revealed that the improvement of the MAS score was positively correlated with the change of ALFF values in the right cerebellum (*r* = 0.698, *P* = 0.001) and the right SMA (*r* = 0.700, *P* = 0.001) after LF-rTMS combined with rPMS treatments (Figure 4). However, no significant correlation between the improvement in MAS scores and the change in ALFF values in other brain areas after LF-rTMS combined with rPMS treatments was noted.

## Discussion

The study trial was designed to explore the effect of the transcranial combined with peripheral repetitive magnetic stimulation on stroke patients with upper limb spasticity. We found that LF-rTMS combined with rPMS improved the spastic state, motor function, and activity of daily living in PSS patients. Furthermore, these improvements accompanied changes in spontaneous neuronal activity in cerebellar and frontoparietal cortical regions. These results established that transcranial combined with peripheral repetitive magnetic stimulation is therapeutically effective for PSS patients, and changes in ALFF values provide different insights into the understanding of the potential neuronal plasticity in LF-rTMS combined with rPMS treatment.

### Improvements in the spastic state and motor function in PSS patients after LF-rTMS combined with rPMS treatment

Maladaptive neural plasticity leads to reticulospinal hyperexcitability and represent be an important mechanism for post-stroke spasticity (Li and Francisco, 2015; Li, 2017). Neuroplasticity can be promoted and modulated through rehabilitation strategies. Following LF-rTMS stimulation of the contralesional cerebral cortex, brain nerves produce plasticity changes that reduce inhibition of the ipsilesional hemispheres and balance interhemispheric activity, facilitating recovery of post-stroke spasticity (Watanabe et al., 2018; Gottlieb et al., 2021). Kakuda et al. (2011) showed that LF-rTMS improves motor dysfunction and relieved upper limb spasticity in stroke patients. Similarly, Li et al. (2021) applied LF-rTMS stimulation to the contralesional M1 area for 4 weeks of treatment, and the results demonstrated that LF-rTMS improved limb motor function and relieved muscle spasticity in stroke patients. Our study also found that spasticity improved after LF-rTMS treatment compared to that noted before treatment, but no significant difference was noted compared with the routine rehabilitation training, which was consistent with previous meta-analysis results (Xu P. et al., 2021).

Moreover, our study found that by employing combined treatment with LF-rTMS and rPMS, the improvements in spasticity, motor function and mobility of patients were more significant than those of the LF-rTMS and control groups. As a non-invasive brain stimulation technique, LF-rTMS can not only induce cortical neuroplasticity and strengthen the connection between cortical neurons, but also temporarily increase cerebral blood flow and improve corticospinal tract function, which is conducive to relieving limb spasticity and promoting motor function rehabilitation (Khedr and Fetoh, 2010; Li Y. et al., 2020; Luk et al., 2022). In addition, rPMS intervention can regulate the proprioceptive delivery nerves, affect proprioceptive and somatosensory input, and strengthen the positive feedback and input of sensory and motor control patterns to the center, which is conducive to improving the motor control of stroke patients (Sakai et al., 2019; El Nahas et al., 2022). Therefore, transcranial magnetic stimulation combined with peripheral magnetic stimulation can stimulate the central and peripheral nervous systems from top to bottom and bottom to top, respectively. This approach can exert a synergistic superposition effect of two stimulations to enhance the downward inhibition of the cortical motor center in the affected side and subsequently inhibit the hyperactive stretch reflex, reducing the degree of limb muscle spasm.

### Changes in brain neural activity after LF-rTMS combined with rPMS treatment

Amplitude of low-frequency fluctuation values are one of the sensitive indicators of rs-fMRI BOLD signals (0.01–0.08 Hz), reflecting the amplitude of spontaneous neural activity in specific regions (Takenaka et al., 2020). Compared with before treatment, the enhanced ALFF following combined treatment included the right SMA and the right middle frontal gyrus. Studies have shown that the SMA region is the main brain functional area controlling sequential movement, and this region participate in the integration of complex movement *via* the fiber connection with the premotor area (PMA), primary motor cortex, and anterior cingulate area (Guehl et al., 2009; Gandolla et al., 2021). The posterior portion of the middle frontal gyrus, the anterior lip of the precentral gyrus, and the superior frontal gyrus together from the PMA are responsible for motor task recognition and behavioral regulation by regulating muscle group movement and posture and maintaining muscle tone (Davare, 2006). During the recovery of motor function in stroke patients, SMA and PMA have significant effects on muscle tension control (Li et al., 2019). Our results showed increased spontaneous neural activity in the SMA and PMA areas of the affected side, suggesting that transcranial combined with peripheral repetitive magnetic stimulation can promote the reorganization of related motor functional areas after stroke. Second, the decreased ALFF in the postcentral gyrus after LF-rTMS combined with rPMS treatment may reflect changes in the internal performance of the subjects’ hands due to reduced input of proprioceptive information from the spastic limb. In brief, our findings suggest that LF-rTMS combined with rPMS can modulate the excitability of the relevant motor cortex and promote the functional reorganization of the cerebral cortex to restore normal activity patterns. These findings are similar to previous studies showing that spasticity relief was associated with changes in neural activation within the frontoparietal cortical region (Struppler et al., 2007).

Furthermore, we compared the changes in ALFF after combination treatment, LF-rTMS, and control treatment. The study found that LF-rTMS combined with rPMS could decrease the ALFF value of left precentral gyrus compared with the routine rehabilitation training group and LF-rTMS group. Functional connectivity of the sensorimotor network and related cortical activation abnormalities may be involved in the occurrence of spasticity. Most studies report that PSS patients show excessive activation of non-affected brain regions before treatment and decreased activation of non-affected brain regions after treatment (Tomášová et al., 2013; Bergfeldt et al., 2015). By reducing the inhibition of the corresponding region of the affected hemisphere and reconstructing the bilateral hemispheric equilibrium state, the spasticity of stroke patients can be relieved (Watanabe et al., 2018). The decreased ALFF in the left precentral gyrus after LF-rTMS combined with rPMS treatment may reflect the notion that combined central and peripheral stimulation seems to promote more cortical excitatory changes to restore normal activity patterns.

Interestingly, the ALFF values of the right cerebellum was increased after LF-rTMS combined with rPMS treatment. The cerebellum is a crucial contributor to motor control. Most studies have shown that dysfunctions of the “cerebellar-thalamic-cortex” pathway composed of various anatomical structures in the basal ganglia, cerebellum and cortex lead to the pathogenesis of spasticity (Shakkottai et al., 2017; Nieuwhof et al., 2022). Additionally, the cerebellum has always been the target of neuromodulation stimulation treatment for spasticity (Li et al., 2021), suggesting that cerebellar activity may be related to the pathophysiology of spasticity. In our study, right cerebellar ALFF values increased after LF-RTMS combined with rPMS treatment, which may be caused by reduced inhibition from Purkinje cell activity associated with decreased afferent discharges. This finding is consistent with a previous study that found that blocking Purkinje cell input with botulinum toxin A injection leads to increased activation in the cerebellum (Chang et al., 2015). Some studies have also shown that the effects of non-invasive nerve stimulation point to cerebellar overactivation, which may counteract cortical deinhibition (Odorfer et al., 2019). Further studies are needed to investigate these mechanisms.

Some limitations were pointed out. In this study, the sample size of patients was relatively small, and the long-term effects of intervention were not assessed. Future studies should include larger sample sizes and assess the long-term effects of treatment. Second, we only analyzed RS-FMRI data. The combination of multimodal neuroimaging and bioinformatics allows for a more complete understanding of therapeutic effects. Future studies may combine multimodal neuroimaging and biomechanics to further explore the underlying mechanism of transcranial combined with peripheral repetitive magnetic stimulation.

## Summary

In conclusion, transcranial repetitive magnetic stimulation combined with peripheral repetitive magnetic stimulation could improve spastic state, motor function, and activity of daily living in PSS patients, and this effect may be associated with altered cerebellar and frontoparietal cortical activity. Our study provides a beneficial supplementary rehabilitation technology for motor neurorehabilitation in stroke patients with muscle spasticity.

## Data availability statement

The original contributions presented in this study are included in the article, further inquiries can be directed to the corresponding author.

## Ethics statement

The studies involving human participants were reviewed and approved by the Ethics Committee of the 900th Hospital of People’s Liberation Army. The patients/participants provided their written informed consent to participate in this study. Written informed consent was obtained from the individual(s) for the publication of any potentially identifiable images or data included in this article.

## Author contributions

YQ and XL created the study and drafted the manuscript. YZ and JW collected the data and did the experiments. XW conducted the MRI data scanning. All authors contributed to the article and approved the submitted version.

## Footnotes

1
http://restfmri.net/

2
https://www.fil.ion.ucl.ac.uk/spm/
